# Healthcare workers’ mental health during covid-19: placing longitudinal findings in a broader context

**DOI:** 10.1192/j.eurpsy.2026.10364

**Published:** 2026-07-31

**Authors:** D. Czepiel

**Affiliations:** 1 Parnassia Psychiatric Institute; 2 Clinical, Neuro- and Developmental Psychology, Vrije Universiteit Amsterdam, Amsterdam, Netherlands

## Abstract

**Introduction:**

Healthcare workers (HCWs) are recognized as a high-risk group for mental health problems. This has been more widely acknowledged since the COVID-19 pandemic, given HCWs’ frontline role and sustained exposure to occupational and pandemic-related stressors. Although numerous cross-sectional studies have reported elevated symptoms among HCWs, longitudinal evidence—particularly studies incorporating pre-pandemic data—and comparisons with other populations remain limited.

**Objective:**

To examine long-term changes in HCWs’ mental health using evidence from studies with pre-pandemic baseline assessments and to contextualize these changes relative to other high-risk and general population groups.

**Methods:**

We synthesized evidence from longitudinal studies including baseline mental health assessments conducted prior to 2020 to examine changes in HCWs’ mental health during COVID-19. To contextualize these findings, we analyzed harmonized data from six European cohorts (n=2,882) from the Netherlands, France and Germany, compiled within the EU-funded RESPOND project. We investigated changes in anxiety and depression symptoms—assessed using different validated scales across cohorts and standardized for comparability—before and during the pandemic among individuals in the general population and those with pre-existing psychiatric conditions, another group at elevated risk for adverse mental health outcomes. Participants were eligible if they had data from at least one assessment conducted before 2020, as well as two assessments during the pandemic.

**Results:**

HCWs’ mental health trajectories indicated symptom worsening during the pandemic. However, substantial variability was observed, including symptom improvement in some subgroups, gender‑specific patterns, an absence of greater deterioration compared with other occupational groups, and more stable trajectories among individuals with higher pre-pandemic symptom levels. In the harmonized cohort analyses, most individuals in the general population exhibited decreasing symptoms over time, including those with higher initial severity. A smaller subgroup maintained consistently low symptoms. Among individuals with pre-existing psychiatric conditions, approximately half improved from moderate symptom levels, whereas the remainder showed persistently high or low symptoms.

**Conclusions:**

Across groups, mental health trajectories reflected both resilience and vulnerability rather than uniform deterioration. HCWs did not consistently show worse long-term outcomes than other groups, emphasizing the role of baseline symptoms and individual heterogeneity. These findings support targeted, flexible mental health support during future public health emergencies.

**Disclosure of Interest:**

None Declared

